# Scabies Mite Inactive Serine Proteases Are Potent Inhibitors of the Human Complement Lectin Pathway

**DOI:** 10.1371/journal.pntd.0002872

**Published:** 2014-05-22

**Authors:** Simone L. Reynolds, Robert N. Pike, Angela Mika, Anna M. Blom, Andreas Hofmann, Lakshmi C. Wijeyewickrema, Dave Kemp, Katja Fischer

**Affiliations:** 1 Infectious Diseases Division, QIMR Berghofer Medical Research Institute, Brisbane, Australia; 2 Department of Biochemistry and Molecular Biology, Monash University, Melbourne, Australia; 3 Diagnostics Development, Bernhard Nocht Institute for Tropical Medicine, Hamburg, Germany; 4 Department of Laboratory Medicine, Lund University, Malmö, Sweden; 5 Structural Chemistry Program, Eskitis Institute, Griffith University, Brisbane, Australia; National Institute of Allergy and Infectious Diseases, United States of America

## Abstract

Scabies is an infectious skin disease caused by the mite *Sarcoptes scabiei* and has been classified as one of the six most prevalent epidermal parasitic skin diseases infecting populations living in poverty by the World Health Organisation. The role of the complement system, a pivotal component of human innate immunity, as an important defence against invading pathogens has been well documented and many parasites have an arsenal of anti-complement defences. We previously reported on a family of scabies mite proteolytically inactive serine protease paralogues (SMIPP-Ss) thought to be implicated in host defence evasion. We have since shown that two family members, SMIPP-S D1 and I1 have the ability to bind the human complement components C1q, mannose binding lectin (MBL) and properdin and are capable of inhibiting all three human complement pathways. This investigation focused on inhibition of the lectin pathway of complement activation as it is likely to be the primary pathway affecting scabies mites. Activation of the lectin pathway relies on the activation of MBL, and as SMIPP-S D1 and I1 have previously been shown to bind MBL, the nature of this interaction was examined using binding and mutagenesis studies. SMIPP-S D1 bound MBL in complex with MBL-associated serine proteases (MASPs) and released the MASP-2 enzyme from the complex. SMIPP-S I1 was also able to bind MBL in complex with MASPs, but MASP-1 and MASP-2 remained in the complex. Despite these differences in mechanism, both molecules inhibited activation of complement components downstream of MBL. Mutagenesis studies revealed that both SMIPP-Ss used an alternative site of the molecule from the residual active site region to inhibit the lectin pathway. We propose that SMIPP-Ss are potent lectin pathway inhibitors and that this mechanism represents an important tool in the immune evasion repertoire of the parasitic mite and a potential target for therapeutics.

## Author Summary

Scabies is a skin infection caused by parasitic scabies mites. There are an estimated 300 million cases globally, with the majority of infections occurring in the world's poorest communities. In Australia, scabies is common in remote Indigenous communities where the infection rate is 16 times higher than the non-Indigenous population. Current treatments have remained relatively unchanged for years and consequently treatment resistance has inevitability emerged. Despite scabies being a well known and frequent infectious skin disease, scabies research has been neglected, resulting in a lack of basic scabies mite biological data. As a result no new therapeutics have been developed. Our research seeks to understand the relationship between the parasite and the human host and one key area of interest is how mites avoid destruction and survive in human skin. We have determined that to survive an attack by the skin's immune defence system the mites release counter defensive proteins that inhibit the skin's defences from activating. This strategy allows the mites to survive in the skin, reproduce and to establish an infection. With this information we can design therapeutics that target these mite proteins, allow the skin to mount an attack and potentially reduce infection.

## Introduction

Scabies is an infectious skin disease caused by the mite *Sarcoptes scabiei* and has been classified as one of the six most prevalent epidermal parasitic skin diseases infecting populations of the world living in poverty by the World Health Organisation [1]. A quintessential feature of the scabies infection is the broken epidermal tissue resulting from the mite burrowing into the host epidermis and patient scratching. The tissue damage and release of antigens and excretory products from the mite trigger activation of host complement components in the burrow, where the mite ingests them [2], [3]. The role of complement, a pivotal component of innate immunity, as an important defence against invading pathogens has been well documented and many parasites have an arsenal of anti-complement defences [4], [5]. To avoid complement-mediated mite gut damage, *S. scabiei* has evolved an intricate set of complement inhibitors [3].

We previously reported on a family of scabies mite proteolytically inactive serine protease paralogues (SMIPP-Ss) thought to be implicated in host defence evasion [3], [6]. Further studies revealed that at least five members of the SMIPP-S family function as complement inhibitors [7]. Representative SMIPP-Ss of each clade within the thirty-three member family were localised to the mite gut and mite faeces in host skin [8]. The co-localisation of complement components in the mite gut makes this is an appropriate location for scabies mite anti-complement molecules [2], [3]. We have since shown that two family members, SMIPP-S D1 and I1 (D1 and I1 respectively), have the ability to bind the complement components C1q, mannose binding lectin (MBL) and properdin and are capable of inhibiting all three pathways [3].

Crystal structures of D1 and I1 revealed that occlusion of the S1 subsite of both the SMIPP-Ss by a conserved tyrosine residue blocked potential protein or peptide substrate access [9]. It was therefore concluded that the SMIPP-Ss had “lost” the ability to bind substrates in a classical fashion and had evolved alternative interaction sites. Screening of a phage display library, used to identify peptide substrates of several chymotrypsin-like serine proteases, found no evidence of binding by the SMIPP-Ss, supporting the above hypothesis [9]. Overlaying of thirty-three SMIPP-S sequences, on the two crystal structures, revealed areas of high conservation containing surface exposed residues, which could represent potential functional sites for protein-protein interaction. Interestingly, the regions of conservation were found to exist not on the side of the surface containing the defunct canonical active site, but on the opposite side of the molecule [9].

Although previous studies have demonstrated that SMIPP-Ss can inhibit the classical, alternative and lectin pathways [3] the focus of this study is the inhibition of the lectin pathway. The recent identification of a novel peritrophin localised in the mite gut that appears to trigger activation of the lectin pathway [2] suggests that this pathway would be a major target for inhibition by scabies mite defence proteins, amongst which are the SMIPP-Ss. To elucidate the inhibitory mechanism employed by these scabies mite proteins, we now investigated the functional interaction of D1 and I1 with lectin pathway components and used mutagenesis studies to identify functionally relevant residues. These studies demonstrated that D1 and I1 are potent inhibitors of the lectin pathway and appear to bind MBL, thereby suppressing the activation of the lectin pathway. Interestingly, these two SMIPP-Ss seem to inhibit lectin pathway activation by different mechanisms, facilitated through conserved lysine residues on the SMIPP-S surface.

## Materials and Methods

### Ethics Statement

Normal human serum (NHS) was prepared from the blood of eight healthy donors. Informed written consent was obtained from all blood donors according to the principles of ethical conduct stated in the “National Statement on Ethical Conduct in Human Research” outlined by the Australian National Health and Medical Research Council. The protocol was approved by the medical ethics committee of the QIMR Berghofer Medical Research Institute.

### Recombinant Protein Expression and Purification

Recombinant SMIPP-S protein expression in *Pichia pastoris* and purification were carried out as previously described [3]. Briefly, mature SMIPP-S protein secreted from *P. pastoris* was purified by hydrophobic interaction chromatography on HiTrap phenyl-Sepharose columns (GE Healthcare) followed by dialysis and ion exchange chromatography on HiTrap SP-Sepharose FF columns (GE Healthcare). Purified protein was concentrated in 10 kDa centrifugal filters (Amicon Ultra, Millipore). Centrifugal flow through material was also collected (microfiltrate) and used as a control in assays. Molecular masses and purity of recombinant proteins were confirmed using SDS-PAGE with Coomassie Blue R-250 staining. The purity of the recombinant proteins used in this study were comparable to the protein produced in previous studies [3].

### Site Directed Mutagenesis

Mutations were introduced in the cDNA sequence of D1 (YvT004A06; GenBank accession no. AY333085) and I1 (Yv6023A04; GenBank accession no. AY333081) by site directed mutagenesis. Residues were substituted with alanine or glutamine as outlined in Table 1. All mutants were cloned into the vector pPICZαA (Invitrogen) using primers outlined in Supporting Information Tables S1 and S2 and constructs were transformed into *P. pastoris* strain KM71H. Mutations in SMIPP-S constructs were confirmed by ABI PRISM BigDye Terminator 3.1. Mutant proteins were expressed, purified and concentrated as described above.

**Table 1:** Mutants used to define functional residues in SMIPP-S complement inhibition.

SMIPP-S	Mutant	Mutation Description	Mutation
D1	D1-K103A	single residue changed to alanine	K103A
D1	D1-K103Q	single residue changed to glutamine	K103Q
D1	Mutant 2	multiple residues changed to alanine	L31A, K103A, K104A, E106A, K225A
D1	Mutant 3	multiple residues changed to alanine	K11A, L31A, K100A, K103A, K104A, E106A, K225A
I1	I1-K108A	single residue changed to alanine	K108A
I1	I1-K108Q	single residue changed to glutamine	K108Q
I1	Mutant 4	multiple residues changed to alanine	K10A, Q11A, K108A

### Protein Preparation for Complement Deposition Assays

Protein samples were buffer exchanged to assay buffer using Zeba Desalt columns (Thermo Fischer Scientific, Australia). The same volume of SMIPP-S microfiltrate was also buffer exchanged and used in the assay as a control (microfiltrate).

### Complement Proteins, Antibodies and Normal Human Serum

Complement proteins and antibodies used were Human MBL (Statens Serum Institute, Copenhagen, Denmark), recombinant MASP-2, anti-MASP-1, anti-MASP-2 (Sapphire Biosciences, Australia) and recombinant MASP-1 (Life Research, Australia). Normal human serum was prepared from the blood of eight healthy donors.

### Complement Deposition Assays

All incubation steps were carried out at room temperature (RT) in 50 µl of assay buffer, except washing and blocking, in 250 µl of solution. Microtitre plates (Maxisorp, Nunc) were incubated overnight at 4°C with coating buffer (50 mM sodium carbonate, pH 9.6), 100 µg/ml mannan (Sigma-Aldrich) and 1% (w/v) BSA (negative control) and incubated with blocking buffer (1% [w/v] BSA in PBS) for 2 hr. To analyse the lectin pathway, 2% (v/v) NHS, was incubated in GVB^2+^ buffer (5 mM veronal buffer pH 7.35, 140 mM NaCl, 0.1% [w/v] gelatin, 1 mM MgCl_2_, 0.15 mM CaCl_2_) for 20 min (for detection of C4b and C3b) or 1 hr (for detection of MBL and C9) at 37°C. NHS was pre-incubated for 15 min at RT with various concentrations of SMIPP-S protein, SMIPP-S microfiltrate or BSA, as a negative control, before addition to the microtiter plate. Complement activation was assessed by detection of deposited complement proteins using antibodies against C4c and C3d (Dako), MBL (R&D Systems) and C9 (Complement Technology) diluted in blocking buffer. After 1 hr incubation with the primary antibody, HRP-conjugated secondary antibodies against IgG (Dako) were diluted in blocking buffer and added for 30 min (for C4b and C3b detection) or 1 hr (for MBL and C9). Bound enzyme was detected using o-1,2-phenylenediamine dihydrochloride tablets (OPD, Dako) in presence of hydrogen peroxide. Absorbance was measured at 490 nm. The absorbance value obtained in the absence of SMIPP-S was defined as 100%. Each incubation step was followed by extensive washing using washing buffer (50 mM Tris-HCl pH 8.0, 150 mM NaCl and 0.1% [v/v] Tween-20). Complement activity for protein samples was calculated against the microfiltrate control (taken as 100%) and BSA was calculated against the PBS control (taken as 100%).

### Direct Binding to Complement Components

All incubation steps were carried out at RT in 50 µl of assay buffer, except washing and blocking in 250 µl of solution. Microtitre plates (Maxisorp, Nunc) were incubated overnight at 4°C with coating buffer (as above) containing SMIPP-S proteins (10 µg/ml or 0–20 µg/ml), mannan (100 µg/ml) (positive control), or 1% (w/v) BSA (negative control). After washing and blocking for 2 hr at RT, wells were incubated for 2 hr at RT with MBL (0–20 µg/ml), rMASP-1 (1 µg/ml) or rMASP-2 (1 µg/ml) in HEPES buffer (50 mM HEPES pH 7.4, 100 mM NaCl, 2 mM CaCl_2_). Bound complement protein was detected by incubation with specific antibody against MBL, MASP-1, or MASP-2 for 1 hr at RT, followed by incubation with HRP-conjugated secondary antibody for 1 hr at RT. Bound HRP was detected as described for the deposition assays.

### Competition Binding with MBL-Associated Serine Proteases in MBL complex

All incubation steps were carried out at RT in 50 µl of assay buffer, except washing and blocking in 250 µl of solution. Microtitre plates (Maxisorp, Nunc) were incubated overnight at 4°C with coating buffer (as above) containing SMIPP-S proteins (0–20 µg/ml) or 1% (w/v) BSA (negative control).

After washing and blocking for 2 hr at RT, wells were incubated for 2 hr at RT with purified MBL (10 µg/ml) in HEPES buffer. MASP-1 or MASP-2 protein was detected by incubation with specific antibody for 1 hr at RT, followed by incubation with HRP-conjugated secondary antibody for 1 hr at RT. Bound HRP was detected as described for the deposition assays.

### Determination of Protein Secondary Structure by Circular Dichroism Spectroscopy

Circular dichroic spectra were acquired on a Jasco J-815 CD/ORD spectrometer at 20°C in a quartz cell of 0.1 cm path length. Data were collected at 0.5 nm intervals with five scans taken and averaged. The protein concentration used was 0.3 mg/ml in 50 mM Tris, pH 7.5.

### Statistical Analysis

Statistical significance was calculated by two-way ANOVA and Bonferroni post test (GraphPad Prism (6), GraphPad Software Inc. USA). Values of p<0.05 were considered significant.

## Results

### Complement Deposition Assays Show the Lectin Pathway Is the Primary Target for SMIPP-Ss

**Figure 1:**
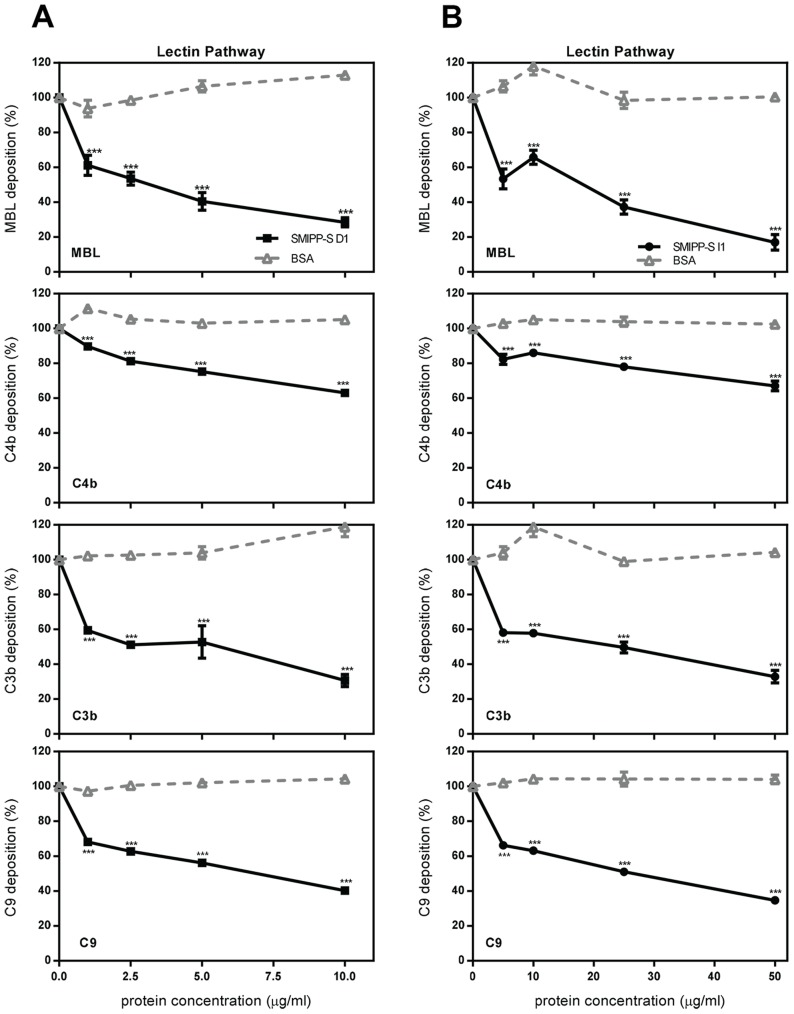
SMIPP-Ss D1 (A) and I1 (B) inhibit the lectin pathway from the level of MBL. Microtitre plates coated with 100 µg/ml mannan were incubated with 2% NHS pre-incubated with various concentrations of SMIPP-S or BSA as a negative control. Complement activation was assessed by detection of deposited MBL, C4b, C3b and C9 with specific antibody. The absorbance value obtained in the absence of SMIPP-S was defined as 100%. The means of three independent experiments performed in duplicate +/− standard deviation (SD) are shown. Statistical significance of observed differences was calculated by two-way ANOVA and Bonferroni post test. *, p<0.05, **, p<0.01, ***, p<0.001, ns, p>0.05.

The deposition of MBL and the downstream components, C4b, C3b and C9 were measured for the lectin pathway following treatment with purified D1 and I1 protein (figure 1). The purity of the recombinant proteins used in this study were comparable to previous studies [3]. D1 inhibited the deposition of MBL from the lectin pathway by 70% at 10 µg/ml, while I1 inhibited MBL deposition by 80% at 50 µg/ml (figure 1A, 1B). The SMIPP-Ss also significantly inhibited deposition of complement components downstream of MBL in the lectin pathway, but to a lesser extent than the impact on MBL deposition. This suggests that the main molecular target for the SMIPP-Ss is MBL.

### The SMIPP-Ss Bind to MBL but Not to MBL-Associated Serine Proteases (MASPs)

**Figure 2:**
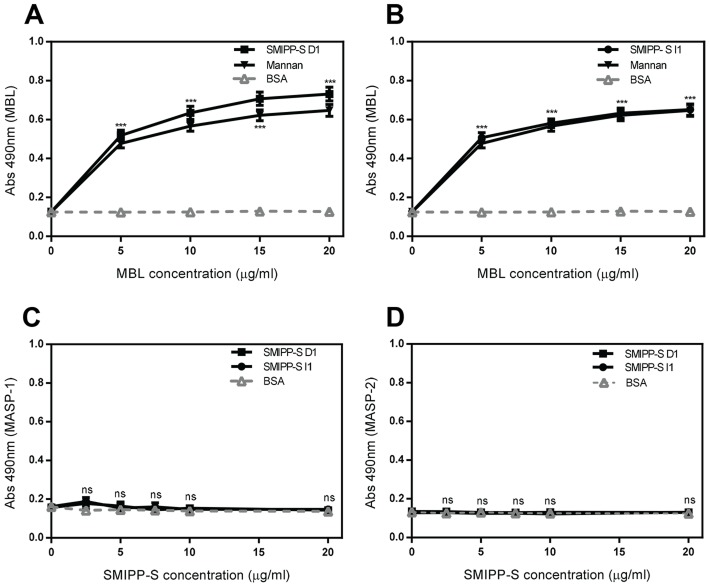
Investigating binding between MBL and MASPs with SMIPP-Ss. To ascertain if SMIPP-Ss bind to MBL when it is associated with MASPs in a complex, immobilised D1 (A) or I1 (B), mannan as a positive control or BSA as a negative control were incubated with increasing concentrations of MBL in complex with MASPSs. MBL was then detected with specific antibody. To determine if SMIPP-S D1 or I1 bind directly to MASP-1 or MASP-2, immobilised SMIPP-S D1, I1 or BSA as a negative control were incubated with rMASP-1 (C) or rMASP-2 (D). rMASP-1 and rMASP-2 were then detected with specific antibody. The means of three independent experiments performed in duplicate +/− standard deviation (SD) are shown. Statistical significance of observed differences was calculated by two-way ANOVA and Bonferroni post test. *, p<0.05, **, p<0.01, ***, p<0.001, ns, p>0.05.

Levels of deposition of the MBL complex were found to be very sensitive to the presence of SMIPP-Ss, thus it is reasonable to expect that these mite proteins might bind to the complex. The complex includes MBL and MBL-associated serine proteases (MASPs). To determine whether the SMIPP-Ss bound to the MBL:MASP complex or to the MASP proteases, direct binding assays were performed. A dose dependent binding to MBL was observed for both the SMIPP-Ss (figure 2A, 2B). However, similar experiments with recombinant MASP-1 and MASP-2 did not show any binding (figure 2C, 2D). These results suggest that the binding of SMIPP-Ss to the MBL complex is mediated via MBL and not the MASP-1 or MASP-2.

### Binding of D1, but Not I1 to the MBL Complex Results in Release of MASP-2

**Figure 3:**
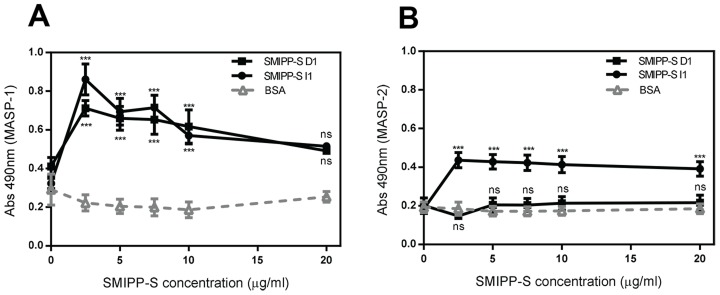
Investigating the mechanism of inhibition of lectin pathway activation via the MBL complex. To determine if MASP-1 (A) or MASP-2 (B) remain associated with MBL in the presence of SMIPP-S D1 or I1 increasing concentrations of immobilised SMIPP-S protein or BSA as a negative control were incubated with MBL complexed with MASPs. MASP-1 and MASP-2 were then detected with specific antibody. The means of three independent experiments performed in duplicate +/− standard deviation (SD) are shown. Statistical significance of observed differences was calculated by two-way ANOVA and Bonferroni post test. *, p<0.05, **, p<0.01, ***, p<0.001, ns, p>0.05.

It is possible that the binding of the SMIPP-Ss to the MBL complex could result in conformational changes, causing either a loss of some functional components (such as the MASPs) or inhibition of the activation of the MASPs in the complex. To elucidate this, we investigated whether SMIPP-S binding results in a release of MASP-1 and/or MASP-2 from the MBL complex. When specific MASP-1 or MASP-2 antibodies were used to monitor integrity of the MBL complex after binding to both the SMIPP-Ss, MASP-1 was still detectable in the complex (figure 3A). In contrast, while MASP-2 was still detectable after I1 treatment, it was absent after D1 treatment (figure 3B). These observations indicate differences in the mechanism of action of the two SMIPP-Ss. Whereas D1 appears to mediate inhibition of the lectin pathway by causing the release of MASP-2 from the complex, I1 apparently inhibits the pathway without causing the release of either protease.

### Mutagenesis Studies Revealed Functional Regions in SMIPP-Ss for Lectin Pathway Inhibition

**Figure 4:**
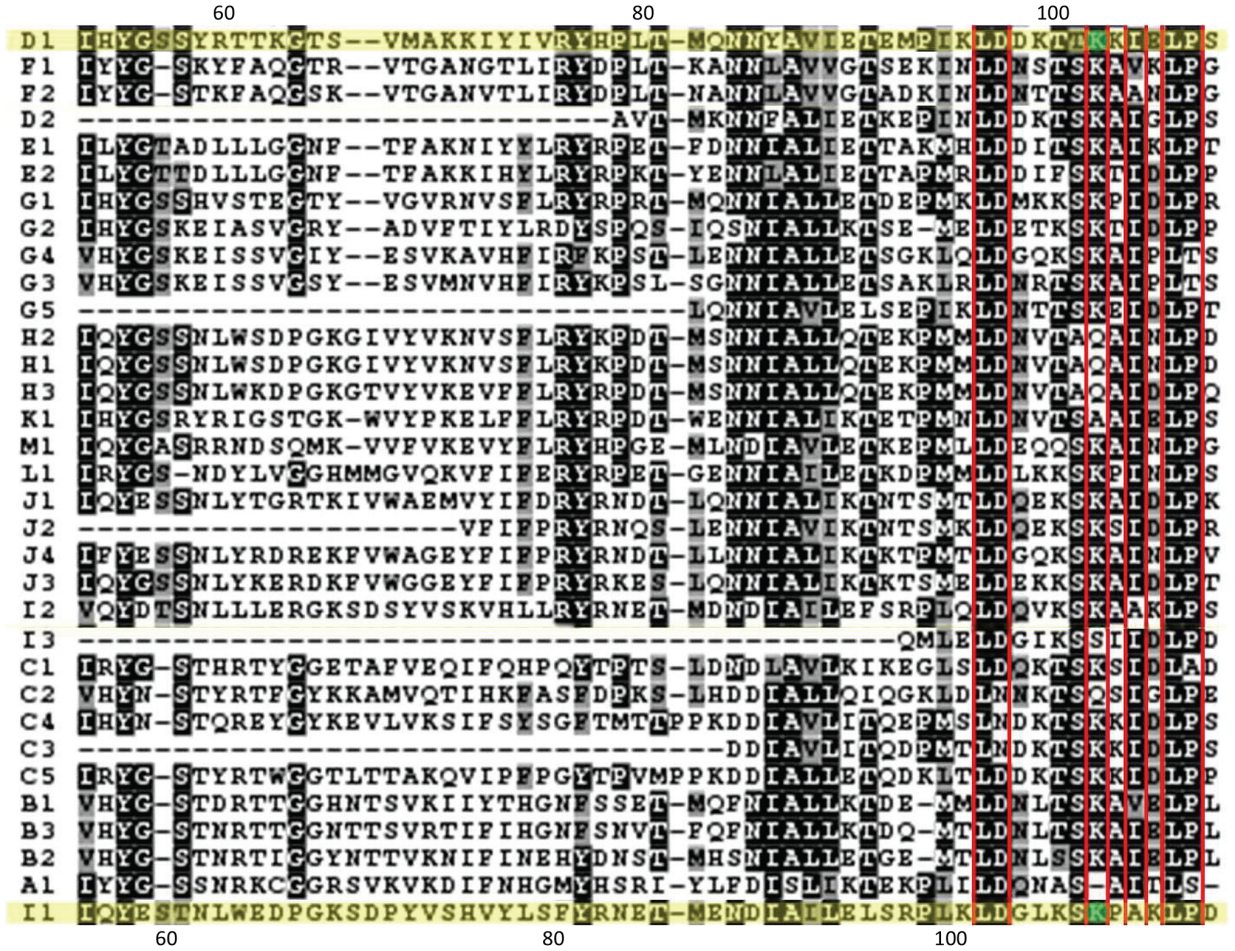
Sequence alignment of the SMIPP-S family. Sequence alignment of the SMIPP-S family highlighted a cluster of conserved surface exposed residues surrounding K103 and K108. Aligned residues are shaded indicating high (black) to low (grey) identity. D1 and I1 sequences are coloured yellow, conserved surface exposed residues are boxed in red and residues K103 and K108 are coloured green.

To pinpoint the MBL binding site on the SMIPP-S molecules, regions of conservation on the SMIPP-S surface containing surface exposed conserved residues were investigated. These regions were previously predicted as potential interaction sites in structural studies [9]. Preliminary mutagenesis studies suggested that residue K103 in D1 and K108 in I1 and the surrounding regions were important for the inhibitory effects on complement (data not shown). Sequence alignment of the thirty-three SMIPP-Ss revealed that residue K103 in D1 and K108 in I1 are conserved surface exposed residues that align in the sequence of all members of the SMIPP-S family (figure 4). Within the tertiary structure, the residue is centrally located within a cluster of conserved surface-exposed residues in its respective SMIPP-S. Focusing on K103 and K108, single point mutations with alanine or glutamine (D1-K103A, D1-K103Q, I1-K108A and I1-K108Q) were used to assess if the residue and/or its charge were functionally relevant in SMIPP-S inhibitory activity. Other conserved and non conserved residues identified in the tertiary structure as being surface exposed and in close proximity to K103 and K108 were also targeted for subsequent mutagenesis studies. Mutants described in this study are shown in Table 1. All substitution mutants were assessed for their ability to inhibit deposition of complement components.

### Analysis of D1 and I1 Mutants Revealed Residues Essential for Lectin Pathway Inhibition

**Figure 5:**
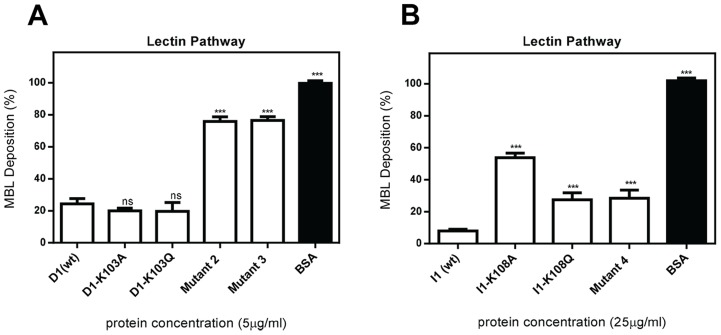
SMIPP-S mutants have lost inhibitory function in the lectin pathway. To determine if D1 mutants (A) and I1 mutants (B) had lost inhibitory function in the lectin pathway deposition assays were performed. Microtitre plates coated with 100 µg/ml mannan were incubated with 2% NHS pre-incubated with 5 µg/ml D1 (positive control) or D1 mutant (A), 25 µg/ml I1 (positive control) or I1 mutant, or BSA as a negative control. Complement activation was assessed by detection of deposited MBL with specific antibody. The means of three independent experiments performed in duplicate +/− standard deviation (SD) are shown. Statistical significance of observed differences was calculated by two-way ANOVA and Bonferroni post test. *, p<0.05, **, p<0.01, ***, p<0.001, ns, p>0.05.

D1 mutants 2 and 3 showed a significant loss of inhibitory activity, as judged by MBL deposition, with no significant differences seen in the other mutants compared to the wild type (figure 5A). All I1 mutants showed a significant reduction in inhibitory activity, with the greatest loss seen in mutant I1-K108A (figure 5B). Collectively these results show that the residues targeted in D1 mutants 2 and 3 and I1 mutants K108A and 4 are involved in preventing the deposition of MBL by these molecules.

To confirm that the secondary structure of the SMIPP-S mutant protein circular dichroism spectroscopy was performed with D1 mutant 2 and I1 mutant 4. These two mutant proteins were chosen as they had yielded the most interesting results for each SMIPP-S and were considered suitable for future assays. Both mutants displayed comparable secondary structure characteristics to the related wild type protein (data not shown). Verification of the integrity of the SMIPP-S structural conformation in the mutants confirmed the functional relevance of the residues targeted for mutation in inhibitory activity.

## Discussion

The recent finding that a mite gut wall peritrophin molecule stimulates the activation of the lectin pathway of complement [2] would suggest that this pathway is the most likely target of mite defence molecules, such as the SMIPP-Ss. The two SMIPP-S proteins, prepared as described here, were found to be strong inhibitors of lectin pathway, yielding 50% inhibition of the deposition of MBL with as little as 5 µg/ml D1 (191 nM) and 15 µg/ml I1 (589 nM). As the *in vivo* concentrations of SMIPP-Ss in the mite gut and complement proteins, such as MBL, at the infection site have yet to be determined, it is difficult to determine how related these concentrations are physiologically. Most likely *in vivo* the mite molecules are highly concentrated at the local level within the confined mite gut and the burrows. The assays shown are done under *in vitro* conditions with recombinant molecules that were expressed and folded *in vitro*. What percentage of the recombinant molecules is biologically active is unknown. Given that, compared to lectin pathway studies with other parasite recombinant MBL inhibitors these concentrations are comparable [10]. The action of the SMIPP-Ss at the level of MBL in the lectin pathway suggested that MBL is a major target and central to the SMIPP-S inhibitory mechanism.

Human MBL is a collagen-like structure comprised of a C-terminal moiety containing carbohydrate recognition domains and a N-terminal moiety containing a collagen-like domain referred to as the stalk [11]. The collagen stalk is the binding site of MASPs, which activate downstream complement components [11]. Given that no direct binding to MASPs was evident, other mechanisms may be operating. Interestingly, in the presence of D1, MASP-2 was no longer detectable in the complex, suggesting that either D1 binds at the same location on the MBL collagen stalk as MASP-2 or in close proximity resulting in the release of MASP-2 from the complex. Release of MASP-2 from the MBL/MASP complex would most likely prevent its activation and subsequent downstream production of the C3 convertase. By contrast, I1 did not release MASP-1 or MASP-2 from the complex. However, the deposition assays demonstrated that lectin pathway activation was inhibited from the level of MBL (figures 1A, 1B). Based on these data it seems reasonable to assume that I1 binds to an undefined site in the MBL collagen stalk, possibly disrupting MBL conformation or access for the MASP substrates. Evidently, the mechanisms employed by the two SMIPP-Ss differ, but both disrupt MASP activation. A preference for collagen binding is supported by previous studies demonstrating direct binding by SMIPP-Ss to the collagen stalk of C1q [3]. Given the high structural and sequence homology shared by MBL and C1q, one can assume that this same region would be the SMIPP-S binding region in MBL.

To further define this binding interaction, mutagenesis studies investigating functional residues involved in lectin pathway inhibition by D1 and I1 were conducted. Preliminary studies identified a specific region on the surface of each SMIPP-S as a potential interaction site. Focusing on these regions, a residue common to both SMIPP-Ss, aligning in the sequence, was identified, K103/K108. Given the initial thoughts that functionally relevant regions and residues could be conserved between the SMIPP-Ss, residues K103 (D1) and K108 (I1) were investigated. To assess residue importance, a single point mutation to alanine or glutamine was introduced at this position and tested in lectin pathway deposition assays.

For D1, the single point mutation to either an alanine or glutamine (D1-K103A, D1-K103Q) residue did not result in the loss of inhibitory activity. The lack of change in the effect on pathway activation suggests that either K103 is not an important functional residue, or that targeting K103 alone was not enough to disrupt the inhibitory activity in D1. For I1, both single point mutations (I1-K108A, I1-K108Q) were shown to have caused a significant reduction of inhibitory activity. Comparative analysis showed that the alanine mutant displayed the greater effect on function. Since a K>A mutation results in a loss of positive charge and any potential side chain hydrogen bond formation, both factors (charge and hydrogen bonding) are functionally relevant for inhibition of the lectin pathway by I1. The I1 results indicate that K108 is a pivotal residue in the binding mechanism for this protein. We then explored whether additional surface exposed residues identified in the tertiary structure as being surface exposed and in close proximity to K103 and K108 were also involved in the binding mechanism. Examination of the D1 structure showed that K103 resides in a ridge-like formation of polar residues on the D1 surface. Mutations targeted the regions flanking K103 along the ridge (mutant 2) and spanning the ridge (mutant 3). Residue K108 also sits in a smaller ridge-like formation hence only two additional residues (K10 and Q11) were mutated.

Results obtained with constructs 2 and 3 confirmed the relevance of the targeted region in D1. Given that the inhibitory activity by mutants 2 and 3 were similar, it would appear that the residues common to these mutants (L31, K104 and K225) may be important in mediating binding to MBL. For I1, the K108 residue appears to be the most important residue since additional substitution of the K10 and Q11 residues in mutant 4 decreased the effect of the K108A mutation on MBL deposition. Future mutagenesis studies investigating the region may help to narrow down critical sites.

Given the above evidence, two potential binding scenarios could be considered: (1) SMIPP-Ss bind to MBL, releasing the MASPs from the MBL complex; or (2) the SMIPP-Ss bind to MBL and confer a disturbance to the conformation of the MBL complex disrupting MASP activation. Binding to MBL could be facilitated through a negatively charged region in the collagen stalk recently identified as being responsible for enhancing phagocytosis [12]. This region is in close proximity to the MASP binding site. Once a MBL/MASP-2 complex is formed, the MASP-2 would autoactivate when MBL binds to ligands [13], [14]. This activation is thought to involve a conformational change in the collagen stalk of MBL, which impacts on the MASP [15], [16]. The SMIPP-S lysine residues may act as a positively charged patch, which can engage with the negatively charged region on the MBL stalk. As the MBL stalk binding region in D1 is larger than I1, the D1 binding region may extend into the MASP binding region. This, in turn, could explain why D1 binding results in release of MASP-2 – a phenomenon, which does not occur with I1. The fact that the binding site for MASP-1 and MASP-2 are not identical explains why only MASP-2 was released [17]. Importantly, either scenario results in a suppression of MASP activation by SMIPP-Ss, and subsequently, suppressed activation of downstream complement components. Given that the inhibitory mode of action appears to differ between the two SMIPP-Ss future studies evaluating if D1 and I1 act synergistically, perhaps even at lower concentrations, will be of great interest. Previous synergistic studies of scabies mite serpins, that are also anti-complement molecules, did demonstrate synergistic activity [18]. If this is the case with the SMIPP-Ss, a multivalent drug design may need to be considered.

This study provides evidence supporting the conclusion that SMIPP-Ss inhibit the lectin pathway through binding to MBL. Notably, the results indicate that the mode of action of individual SMIPP-Ss could be different. A family of proteins that target complement pathway(s) by different mechanisms would represent a highly sophisticated and diverse immune evasion strategy by this parasite. Further studies will help to clarify the roles of SMIPP-Ss in immune evasion and open avenues to the design of novel therapeutics.

## Supporting Information

Table S1 **Primer sequences for SMIPP-S D1 mutants.**
(PDF)

Table S2 **Primer sequences for SMIPP-S I1 mutants.**
(PDF)
