# Supplementary material for: Scabies Mite Inactive Serine Proteases Are Potent Inhibitors of the Human Complement Lectin Pathway
Source: PLoS Negl Trop Dis. 2014 May 22;8(5):e2872. doi: 10.1371/journal.pntd.0002872 (PMC4031079; doi:10.1371/journal.pntd.0002872)
Supplement: Table S1 — Primer sequences for SMIPP-S D1 mutants. (PDF) [file pntd.0002872.s001.pdf]

Table S1: Primer Sequences for SMIPP-S D1 Mutants

Mutant	Primer	Sequence 5' – 3'
D1-A	K103A For	GGATGATAAGACAACCGCAAAAATAGAATTGCC
	K103A Rev	GGCAATTCTATTTTTGCGGTTGTCTTATCATCC
D1-Q	K103Q For	GGATGATAAGACAACCCAAAAAATAGAATTGCC
	K103 Rev	GGCAATTCTATTTTTTGGGTTGTCTTATCATCC
Mutant 2	L31A For	GCGGGAGCATTGCAACTGCAAACTTTG
	L31A Rev	CAAAGTTTGCAGTTGCAATGCTCCCGC
	K104A E106A For	GATAAGACAACCGCAGCAATAGCATTGCCATCATTGCTC
	K104A E106A Rev	GAGCAATGATGGCAATGCTATTGCTGCGGTTGTCTTATC
	K225A Rev	ACCGGCGGCCGCTCAATATTCTTGAGGTTTAACTTTGCCACTGATAATATCTGCGATATC
Mutant 3	K11A For	ACCGCTCGAGAAAAGAATCATAGGTGGTAAGAAGAGTGATATCACTGCAGAACCATGG
	K100AK104AE106AFor	ATAAAGTTGGATGATGCGACAACCGCAGCAATAGCATTGCCATCATTGCTC
	K100AK104AE106ARev	GAGCAATGATGGCAATGCTATTGCTGCGGTTGTCGCATCATCCAACTTTAT
	K225A Rev	ACCGGCGGCCGCTCAATATTCTTGAGGTTTAACTTTGCCACTGATAATATCTGCGATATC
D1	D1 Pichia For	ACCGCTCGAGAAAAGAATCATAGGTGGTAAGAAGAGTGATATAACTAAAGAACC
D1	D1 Pichia Rev	ACCGGCGGCCGCTCAATATTCTTGAGGTTTAACTTTGCCACTGATAATATC
