# Supplementary material for: Scabies Mite Inactive Serine Proteases Are Potent Inhibitors of the Human Complement Lectin Pathway
Source: PLoS Negl Trop Dis. 2014 May 22;8(5):e2872. doi: 10.1371/journal.pntd.0002872 (PMC4031079; doi:10.1371/journal.pntd.0002872)
Supplement: Table S2 — Primer sequences for SMIPP-S I1 mutants. (PDF) [file pntd.0002872.s002.pdf]

Table S2: Primer Sequences for SMIPP-S I1 Mutants

Mutant	Primer ID	Sequence 5' - 3'
I1-A	K108A For	GGATGGACTCAAAAGTGCACCTGCTAAATTGCC
	K108A Rev	GGCAATTTAGCAGGTGCACTTTTGAGTCCATCC
I1-Q	K108Q For	GGATGGACTCAAAAGTCAACCTGCTAAATTGCC
	K108Q Rev	GGCAATTTAGCAGGTTGACTTTTGAGTCCATCC
Mutant 4	K10A Q11A K108A For	ACCGGTCGACAAAAGAATCAAAGGAGGTGAAAAAACCGACATCGCAGCAGTACCTTGG
I1	I1 Pichia For	ACCGGTCGACAAAAGAATCAAAGGAGGTGAAAAAACCGACATCAAACAAG
I1	I1 Pichia Rev	ACCGGCGGCCGCTTATGATTTCTTCTTAATTATATCTTGTATCCATGA
